# Supplementary material for: RAD18 opposes transcription-associated genome instability through FANCD2 recruitment
Source: PLoS Genet. 2022 Dec 8;18(12):e1010309. doi: 10.1371/journal.pgen.1010309 (PMC9767342; doi:10.1371/journal.pgen.1010309)
Supplement: S1 Table — Table A. Primer sequences from this study. Table B. Plasmids from this study. (DOCX) [file pgen.1010309.s007.docx]

**Table A**. Primer sequences from this study.

| Primer | Sequence | Reference |
| --- | --- | --- |
| Actin Control Fwd | CGGGGTCTTTGTCTGAGC | Hamperl et al., 2017 (18) |
| Actin Control Rev | CAGTTAGCGCCCAAAGGAC | Hamperl et al., 2017 (18) |
| oriP for mAIRN and ECFP plasmid copy number Fwd | TTTTCGCTGCTTGTCCTTTT | Hamperl et al., 2017 (18) |
| oriP for mAIRN and ECFP plasmid copy number Rev | CATTTTCGTCCTCCCAACAT | Hamperl et al., 2017 (18) |
| ECFP Fwd | TGGTTTGTCCAAACTCATCAA | Hamperl et al., 2017 (18) |
| ECFP Rev | ACATGGTCCTGCTGGAGTTC | Hamperl et al., 2017 (18) |
| NRG3 5' Fwd | GAGGCTAGCACCAAGGTCTG | Okamoto et al., 2018 (44) |
| NRG3 5' Rev | ACTGGCTGAGGCTACCAGAA | Okamoto et al., 2018 (44) |
| NRG3 peak Fwd | GAGGCCCAGGACACATAGAA | Okamoto et al., 2018 (44) |
| NRG3 peak Rev | GCAACAGGCTAACATGCAGA | Okamoto et al., 2018 (44) |
| NRG3 3' Fwd | TGTTGCGAAGAACCTGTGAG | Okamoto et al., 2018 (44) |
| NRG3 3' Rev | ACTCCTATGGCTCTGCTGGA | Okamoto et al., 2018 (44) |
| WWOX Fwd | CAGCCAGCACTCCTTCTCAA | Okamoto et al., 2018 (44) |
| WWOX Rev | CTCTGTGGAGAAGCCAAGCA | Okamoto et al., 2018 (44) |
| CDH13 Fwd | CGGGAGACATCCTCGAAATA | Okamoto et al., 2018 (44) |
| CDH13 Rev | CCTAGCACCAGGTCTTGCTG | Okamoto et al., 2018 (44) |
| LRP1B Fwd | GCCTATACCGGAAGACTGTGTG | Okamoto et al., 2018 (44) |
| LRP1B Rev | GGCTGCTCAGAAGACCACTTTA | Okamoto et al., 2018 (44) |
| APOE Fwd | GGGAGCCCTATAATTGGACAAGT | Barroso et al., 2019 (29) |
| APOE Rev | CCCGACTGCGCTTCTCA | Barroso et al., 2019 (29) |
| RPL13A Fwd | GCTTCCAGCACAGGACAGGTAT | Barroso et al., 2019 (29) |
| RPL13A Rev | CACCCACTACCCGAGTTCAAG | Barroso et al., 2019 (29) |
| MIB2 Fwd | CTCTCCTTGTCTGGGGCTC | Barroso et al., 2019 (29) |
| MIB2 Rev | CTGCCTCCCTCACCTGTC | Barroso et al., 2019 (29) |
| RHOT2 Fwd | GTGCCAGGCTGTATTGCTT | Barroso et al., 2019 (29) |
| RHOT2 Rev | GGGAAATGCAGACGTGTCAT | Barroso et al., 2019 (29) |

**Table B**. Plasmids from this study.

| Plasmid | Summary | Reference |
| --- | --- | --- |
| ECFP-HO | Expressing ECFP gene with promoter facing towards unidirectional origin of replication | Hamperl et al., 2017 (18) |
| ECFP-CD | Expressing ECFP gene with promoter facing away from unidirectional origin of replication | Hamperl et al., 2017 (18) |
| mAIRN-HO | Expressing mouse AIRN gene with promoter facing towards unidirectional origin of replication | Hamperl et al., 2017 (18) |
| mAIRN-CD | Expressing mouse AIRN gene with promoter facing away from unidirectional origin of replication | Hamperl et al., 2017 (18) |
| pRAD18 | Expresses c-myc-tagged human RAD18 | Watanabe et al., 2009 (40) |
| p6BD | Expresses c-myc-tagged human RAD18 deleting hRAD6 binding domain | Watanabe et al., 2009 (40) |
